# "In-house" versus "home" rehabilitation programme following cardiac surgery: A service evaluation

**DOI:** 10.1186/1749-8090-10-S1-A54

**Published:** 2015-12-16

**Authors:** Annemarie Brunswicker, Shakil Farid, Nicola Cooper, Vicky Hatch, Abu-Omar Yasir

**Affiliations:** 1 Papworth Hospital, Papworth Everard, CB23 3RE, UK

## Background/Introduction

Cardiac rehabilitation is the process by which patients with cardiac disease are supported to achieve optimal physical and psychosocial health. Exercise training is a core element of cardiac rehabilitation. At our institution, there are two different rehabilitation programmes available to patients who have undergone cardiac surgery: Firstly, there is the "in-house" (IH) hospital-based programme, secondly, the "Road to Recovery" (R2R) home programme.

## Aims/Objectives

Our objective was to compare patient results and potential to improve exercise tolerance after cardiac for both rehabilitation programmes.

## Method

We included all patients entering either rehabilitation program following cardiac surgery from January 2012 to date. We recorded patient demographics and patients' performance at their first and final exercise session. Metabolic equivalents (METS), minutes performed, Maximum and resting heart rate and rate-pressure-product were noted.

## Results

134 patients were enrolled into the IH program, 32 patients chose the R2R program. Among the IH patients there were 108 (81%) male and 26 female (19%); among the R2R program patients there were 23 male (71%) and 9 female (28%).

There was no significant difference between the groups with regards to the METS pre-rehab (p 0.47). Patients in both groups increased their exercise capacity significantly during the rehabilitation process when the METS and maximal age adjusted predicted heart rate before and after the rehabilitation process were compared. The IH group also showed significantly improved resting heart rate, RPP, length of exercise tolerated (mins).

## Discussion/Conclusion

In summary, both rehabilitation pathways seem to equally improve fitness after cardiac surgery and our data indicates that the home R2R rehabilitation model is equivalent to the hospital-based programme. After cardiac surgery, all patients should be offered the possibility of taking charge of their own rehabilitation at home. This should be done with telephone mentoring and follow-up from a rehabilitation centre. Such a programme would avoid travel cost and time for patients as well as motivating them to take their progress into their own hands.

